# Catch-up growth of infants born to mothers with autoimmune rheumatic disorders

**DOI:** 10.1186/s12969-022-00667-w

**Published:** 2022-02-02

**Authors:** Soo Yeun Sim, Hye Yeon Choi, Min Ho Jung, Soo Young Lee, Jung Woo Rhim, Hyun Mi Kang, Dae Chul Jeong

**Affiliations:** Department of Pediatrics, College of Medicine, Seoul St. Mary’s Hospital, The Catholic University of Korea, 222, Banpodaero, Seocho-gu Seoul, 06591 Republic of Korea

## Abstract

**Background:**

In women with autoimmune rheumatic disorders (ARD), pregnancy complications or postpartum events are more frequent compared to the general population. Transplacental autoantibodies or cytokines influence various fetal and neonatal outcomes. We compared the growth patterns of babies born to mothers with ARD versus healthy mothers to assess the long-term growth outcomes of children born to women with ARD.

**Methods:**

This was a retrospective age-matched cohort analyses of babies born to mothers with ARD from the hospitals belonging to the Catholic University of Korea between 2010 and 2017. Demographic and autoimmune laboratory test data of the mothers and newborns were assessed. Neonatal growth was measured in terms of height and weight, measured at birth and follow-up examinations.

**Results:**

We enrolled 142 infants from mothers with ARD and 149 infants from healthy mothers. There was no significant difference between mothers with ARD and healthy mothers in terms of delivery age, parity, abortion, and premature delivery history. The mothers with ARD were diagnosed with systemic lupus erythematosus (81%), Sjogren syndrome (6%), and other autoimmune phenomena (11%). The groups were significantly different in terms of neonatal characteristics such as prematurity, gestational age, birth weight, and height, but not in Apgar score and delivery type. For most neonates, autoimmune laboratory results were normalized within 1 year, except for anti-La/SSB antibody, which remained high in some. The height and weight for age z-score were lower than the normal age groups at birth but showed catch-up growth by 2 years of age.

**Conclusions:**

Low birthweight and prematurity at birth for neonates born to mothers with ARD could be caught up by 2 years of age, and maternal ARD does not affect the growth of their offspring.

## Background

Autoimmune rheumatic disorders (ARD), including systemic lupus erythematosus (SLE), mostly affect women of childbearing age [1, 2]. Women with ARD are at high risk for pregnancy complications such as prematurity, abortion, severe preeclampsia, and poor neonatal outcomes due to angiogenic problems or higher proinflammatory cytokines [3–7].

In women with ARD, higher pregnancy complications or postpartum events are more frequent compared to the general population [3, 5, 8]. Transplacental autoantibodies or cytokines influence various fetal and neonatal outcomes of those born to mothers with ARD [3, 5, 7, 9]. Autoantibodies that pass through the placenta affect fetal growth and development, and contribute to inducing perinatal outcomes, including congenital heart block or neonatal lupus [3, 5, 6]. Babies born to mothers with SLE may are more likely to have premature birth, be small for gestational age, and have low Apgar score, neonatal lupus, or congenital heart block [4, 5, 10]. Since up to 30% of SLE pregnancies are associated with fetal complications such as fetal growth restrictions and small for gestational age (SGA) babies, growth and development of these babies are an important issue [11].

Children born small for gestational age (SGA) showed catch-up growth during the first 2 years of life, although premature SGA babies born under 32 weeks of gestation had a risk of not catching-up compared to full-term SGA babies [12, 13]. Severe maternal morbidity negatively affects the growth and neurodevelopment of infants [14]. Nevertheless, children born very preterm showed normal weight and height before puberty [15]. In particular, children born to women with SLE showed neurocognitive development problems, including learning disorder or attention deficit [16]. However, up to 24 months of age, there were no abnormal outcomes related to the growth of babies born to mothers with well-controlled SLE during pregnancy [10, 17].

In this study, we compared the growth patterns of babies born to mothers with ARD versus healthy mothers to assess the long-term growth outcomes of children born to women with ARD.

## Methods

### Design

This was a retrospective cohort study investigating the growth outcomes of babies born from mothers with ARD compared to healthy women without any underlying diseases. The medical records were retrospectively reviewed, and demographic characteristics compared. The transplacental autoantibodies were assessed according to infant growth.

### Participants

The inclusion criteria for subjects were babies born from 1) mothers diagnosed with ARD during January 2010 to December 2017 at Seoul St. Mary’s Hospital and Yeouido St. Mary’s Hospital, and 2) gestational age-matched babies born from healthy women without any underlying diseases during the same period. A total of 291 neonates were enrolled as study participants; 142 infants born to mothers with ARD and 149 gestational age-matched babies from the 4,561 infants born to healthy mothers. The study was approved by the Institutional Review Board of the hospitals of the Catholic University of Korea (XC20WIDI0037K).

### Definitions and randomization

Mothers with ARD were defined in this study using the International Classification of Disease (ICD-10) classification (P00.8) as ARD group. Gestational age-matched babies born to healthy mothers (Z37.0, Z380, P03) during the study period were defined as a control group.

Normal babies from healthy mothers were randomly selected according to gestational age matched cases for comparison with babies born to mothers with ARD. Babies that expired prior to 2 years of age from congenital condition such as genetic diseases and monogenic lupus were excluded from the study.

### Data collection

Demographic data of mothers included age at delivery, parity, history of abortion and prematurity, and underlying disease in patients with autoimmune disorders. Newborn infants were compared for sex, prematurity, gestational age, delivery type, birth weight, birth height, and Apgar score at 1 and 5 min. We considered birth events significant if the Apgar score was less than 7 at 1 and 5 min. The babies from mothers with ARD were investigated for the immunological parameters several days after birth, including antinuclear antibody (ANA), anti-double stranded DNA antibody (Ab), anti-Ro/SSA Ab, anti-La/SSB Ab, C3 and C4 levels.

Follow-up growth measurements of babies in both groups were retrieved at birth and at 1, 2, 5, 8, 12, and 24 months. At each time point, the age of premature infants was corrected for gestational age. Initial height and weight at birth and follow-up growth parameters were retrieved through the outpatient clinic setting when visits were made for routine child health examinations and vaccinations. Depending on the vaccination schedule, the measurement times varied from 1 − 2 months for each subject. Babies born to mothers with ARD were studied for follow-up autoantibody levels, if possible, at around 8 months of age. The growth parameters of premature babies were adjusted for corrected age. We calculated adjusted z-scores for postnatal weight and height of babies from both groups using the Korean National Growth Charts 2017 [18].

### Statistical analysis

Statistical analyses were performed using SPSS (Statistical Package for the Social Sciences) software (ver. 25.0; SPSS Inc., U.S.). Student’s t-test was used to compare continuous variables, while the chi-square test was used to assess qualitative variables. The Cochran-Armitage trend test and linear mixed regression model were carried out to find the z-score differences at each time point. Statistical significance was set at *P* < 0.05.

## Results

### Neonatal characteristics

The sex, maturity, gestational age, mean birth weight, mean birth height, delivery type, and Apgar scores of babies from the two groups were compared (Table 1). There were no significant differences in sex or Apgar scores between the two groups. However, ARD group was more likely to be premature, born at an earlier gestational age, born via cesarean section, and shorter and lighter at birth than control group.

**Table 1:** Demographic Characteristics of Infants between from Mother with Autoimmune Disorders and Healthy

	**ARD group (*n* = 142)**	**Control group (*n* = 149)**	*P* value
	N (%)	N (%)	
Male:female ratio	65:77	83:66	.09
Prematurity	39 (27.5)	26 (17.4)	.04
Gestational Age (weeks), mean ± SD	37.0 ± 2.4	37.9 ± 2.3	< .01
Birth weight (kg), mean ± SD	2.665 ± 0.616	3.026 ± 0.60	< .01
Birth height (cm), mean ± SD	47.64 ± 5.53	49.75 ± 3.09	< .01
Cesarean section	90 (66.7)	82 (55.4)	.05
Apgar score < 7 (1 min)	22 (16.3)	25 (16.8)	.91
Apgar score < 7 (5 min)	8 (5.9)	7 (4.7)	.65

Abbreviation: *SD* Standard deviation

Table 2 summarizes the changes in autoimmune laboratory data in ARD group. Follow-up laboratory data were collected at approximately 8 months of age. At birth, about half of these babies present autoantibodies, including ANA (55.2%), anti-Ro/SSA (46.7%), and low complement levels. During the follow-up period, most laboratory results were normalized, except for anti-La/SSB Ab levels, which remained slightly above the normal range for 5.6% of the neonates. No severe complications, such as congenital heart block or neonatal events, including hematologic manifestations, were reported from the study participant during the first two years after birth.

**Table 2:** Changes of Autoimmune Laboratory Values in Babies from Mothers with Autoimmune Disorders

	**At birth**	**Follow up**	*P* value
	N (%)^a^	N (%)^a^	
ANA	68/123 (55.2)	4/34 (11.7)	< .01
Anti-Ro/SSA	21/45 (46.7)	2/18 (11.1)	< .01
Anti-La/SSB	4/46 (8.7)	1/18 (5.6)	.56
Anti-dsDNA	16/111 (14.4)	NA	.02
C3 below normal level	55/116 (47.4)	0/27 (0)	< .01
C4 below normal level	57/116 (49.1)	1/27 (3.7)	< .01

^a^ The number of subjects tested is different for each autoantibody

Abbreviation: *ANA* Anti-nuclear antibody, *SSA* Sjogren syndrome A, *SSB* Sjogren syndrome B, *C3* Complement 3, *C4* Complement 4, *NA* Not applicable

### Maternal characteristics

The average (SD) age at delivery was 33.4 (3.28) years for mothers with ARD and 33.5 (4.2) years for healthy mothers. The parity (49.6% vs. 48.0%, respectively), abortion history (29.9% vs. 27.7%, respectively), and premature birth history (26.6% vs. 17.4%, respectively) of the two groups showed no significant differences (Table 3).

**Table 3:** Demographic Characteristics of Mothers

	**ARD group** **(*n* = 142)**	**Control group** **(*n* = 149)**	*P* value
	N (%)	N (%)	
Age (years), mean ± SD	33.43 ± 3.28	33.52 ± 4.215	.87
Primipara (%)	70 (49.3)	71 (47.7)	.79
Abortion history (%)	41 (28.9)	41 (27.5)	.68
Premature birth history (%)	38 (26.8)	26 (17.4)	.06

Abbreviation: *SD* Standard deviation

The underlying diseases of mothers with autoimmune disorders were also analyzed (Table 4). The most common diagnosis was SLE (81%), followed by Sjogren syndrome (6%), anti-phospholipid Ab positivity (4%), and others including autoimmune hepatitis, juvenile idiopathic arthritis, Sicca syndrome, dermatomyositis, and Raynaud’s syndrome (4%).

**Table 4:** The Characteristics of Mothers with Autoimmune Disorders

Underlying diseases	N (%)
SLE	115 (80.9)
Anti-cardiolipin antibody positive	2 (1.4)
Anti-phospholipid antibody positive	6 (4.2)
Rheumatoid arthritis	1 (0.07)
Behcet disease	1 (0.07)
Sjogren syndrome	9 (6.3)
ANA positive	2 (1.4)
Others (autoimmune hepatitis, JIA, Sicca syndrome, dermatomyositis, Raynaud's syndrome)	5 (3.5)

Abbreviation: *ANA* Anti-nuclear antibody, *JIA* Juvenile idiopathic arthritis, *SLE* Systemic lupus erythematosus

### Growth assessment

The total number of subjects with growth parameters available at follow-up in ARD group were as follows: 49 (2 months), 48 (5 months), 45 (8 months), 38 (12 months), and 22 (24 months), respectively.

**Fig. 1:**
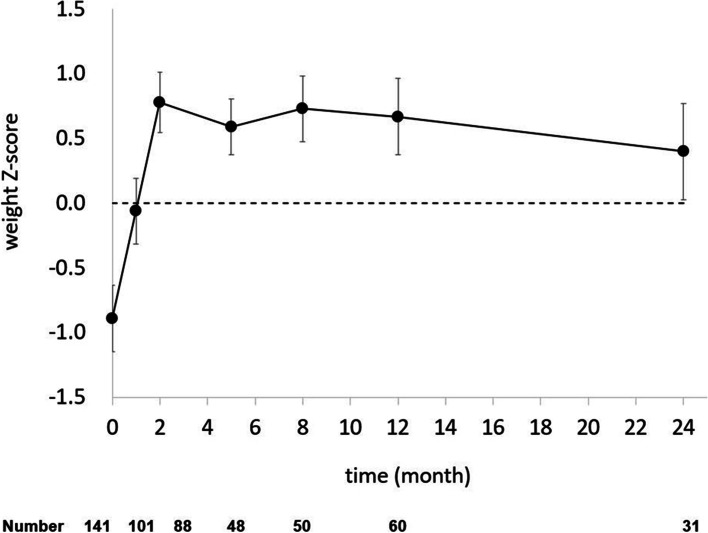
Z-score changes of weight for babies from mothers with autoimmune disorders. The numbers of subjects at each follow-up are shown below

**Fig. 2:**
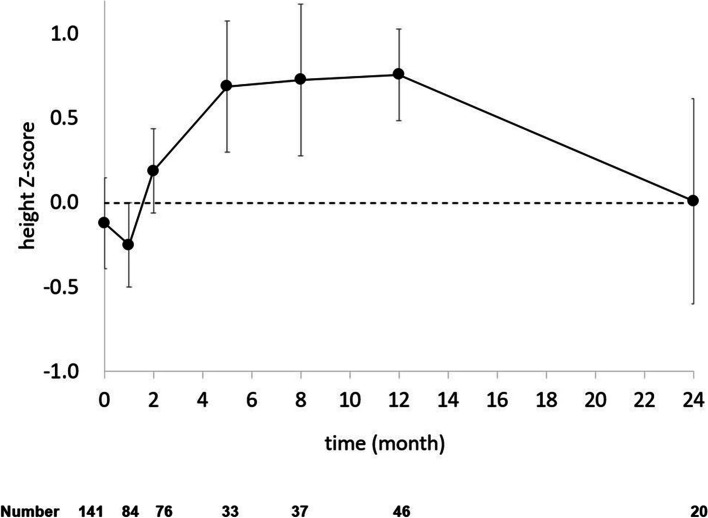
Z-score changes of height for babies from mothers with autoimmune disorders. The numbers of subjects at each follow-up are shown below

In comparison with Korean general population, the height-for-age z-score curve in ARD group is shown in Fig. 1. The height z-score was lower than the normal at 1 month of age, exceeded the average after 2 months, then recovered to normal by 24 months of age. Figure 2 shows the z-score for the weight-for-age of ARD group. At baseline, the mean weight z-scores were –0.83, significantly lower than those in the Korean general population. However, the weight of these infants recovered by 24 months of age.

Since the majority of maternal underlying disease was SLE (81%), we additionally compared growth parameters of babies born to mothers with SLE and other ARD group (data not shown). No significant differences in height and weight of babies of both subgroups, so we assessed the growth parameters of babies born to mothers with ARD including SLE.

## Discussion

As maternal morbidity is known to influence fetal or neonatal outcomes [14], serious perinatal events or growth and neurodevelopment of their babies are a concern for many parents [19]. In this study, we noted catch-up growth in weight and height by 2 years of age in infants born to mothers with ARD, although they showed poorer perinatal outcomes including SGA and prematurity compared to those born to healthy mothers. In addition, autoantibodies present at birth waned during infant growth several months later.

In our study, neonates born to mothers with ARD had a higher rate of prematurity and low birth weight and height. These results are consistent those of previous studies that investigated the perinatal outcomes in children of mothers with SLE [5, 6, 11]. The higher rate of SGA birth noted among infants born to mothers with ARD compared to healthy controls was also compatible with previous study results [6, 20, 21]. SGA in the babies born to mothers with ARD might result from the high level of intra-uterine effects of proinflammatory cytokines or mother’s autoantibody on neonates [3–5, 7]. Many studies showed lower Apgar scores and fetal loss in mothers with ARD than in healthy mothers [4, 10, 22]. However, our results showed that abortion and Apgar scores below 7 were not different between groups. The difference in our study compared to previous findings may be due to differences in disease control during pregnancy in the study population. Moreover, although other studies reported high fetal loss or low Apgar scores in meta-analyses or nation-wide studies [10, 22], only a limited number of cases were included in our study. In pregnancy with ARD, disease control including disease activity may be important to maintain and for favorable perinatal outcome [17, 21, 23].

As the infants grew up, transplacental autoantibodies disappeared during the follow-up period, except for anti-La/SSB Ab. Although infants born to mothers with ARD had autoantibodies, including anti-Ro/SSA Ab, there were no neonatal lupus manifestations including congenital heart block in the neonatal screening or follow-up period. Anti-Ro/SSA and/or anti-La/SSB are known to contribute to congenital heart block or neonatal lupus development [3, 6, 10, 22, 24, 25]. While transplacental autoantibodies gradually diminished 6 months after birth, in this study, anti-SSB antibody remained positive until around 8 months of age compared to other autoantibodies in some children born to mothers with ARD. The presence and titers of maternal anti-Ro/SSA antibodies gradually decreased after birth, they were found until 9 months old [24]. The autoantibodies transferred from mothers with ARD may induce various clinical manifestations of neonatal lupus, and these clinical findings seem to gradually improve up to 12 months of age, owing to the disappearance of maternal autoantibodies [24, 26, 27].

In this study, among the enrolled mothers with ARD, some represented only positive autoantibodies, including ANA, anti-cardiolipin antibody, and anti-phospholipid antibody, even when they were diagnosed with ARD. Pregnancy represents a tolerating immune state due to paternal alloantigen presented by fetal tissue. Sex hormones and the placenta contribute to pregnancy maintenance by modifying T cell function, but changes in T cell immunity during pregnancy might be changed to produce autoantibodies [28]. However, transplacental autoantibodies may affect the fetus by the pathogenesis of autoantibodies [5, 9].

Our results suggest that catch-up growth in infants from ARD mothers took place around 2 years of age in comparison with a normal Korean baby. The z-scores of body weight and height progressively increased as the infants grew. Body weight increased up to 2 years of age in the present study. Lower body weight in infants born to mothers with ARD might be due to poor placental development and persistent clinical or subclinical inflammation with high levels of cytokines or autoantibody reaction [3–7]. Body weight rapidly increased up to 2 months of age, and the z-score was higher than that in normal healthy Korean children until 2 years of age. Rapid catch-up body weight gain occurred at around 3 months of age, followed by a gradual increase. These mechanisms might be due to loss of placental function after birth, and limitation of autoantibody reaction by disruption between baby and mother [4, 7, 9]. However, the z-score for height decreased up to 1 month, and then rapidly increased over 2 years, showing a nearly normal z-score at 2 years of age. Some babies showed lower weight and height at 3 and 9 months of age, but their final height and weight were normal [21]. Postnatal growth in full-term and preterm SGA infants showed catch-up growth at 2 years of age [12, 13].

As our study included a relatively small number of subjects compared to other studies that assessed national data, further large scale studies are needed. Moreover, as we retrospectively reviewed patients’ charts, we were not able to obtain measurements at every time point for each neonate. Other developmental factors were not included in this study and require further investigation. We were unable to investigate more specific maternal demographics, such as disease activity and treatment received. The effect of maternal therapy on offspring’s growth development may provide more insight into the impact of maternal disease on neonatal outcomes [3, 11, 22, 26].

## Conclusions

This study is the first longitudinal study to assess the growth parameters of infants born to mothers with ARD. Our results suggest that weight and height differences could be caught up by 2 years of age in children born from mothers with ARD, and most transplacental autoantibodies could be normalized as neonates grow up. This study favorably suggests that maternal ARD may not affect growth for up to 2 years.

## Data Availability

The datasets used and/or analyzed during the current study are available from the corresponding author on reasonable request.

## Abbreviations

ARD: Autoimmune rheumatic disorders
SLE: Systemic lupus erythematosus
SGA: Small for gestational age
Ab: Antibody
ANA: Anti-nuclear antibody
SSA: Sjogren syndrome A
SSB: Sjogren syndrome B
C3: Complement 3
C4: Complement 4
